# Water-Immersible MEMS Mirror with a Large Optical Aperture

**DOI:** 10.3390/mi15020235

**Published:** 2024-02-02

**Authors:** Yi Yang, Yichen Liu, Yongquan Su, Yang Wang, Yonggui Zhang, Hao Chen, Lihao Wang, Zhenyu Wu

**Affiliations:** 1 School of Microelectronics, Shanghai University, Shanghai 200444, China; 2 Shanghai Industrial Technology Research Institute, Shanghai 201800, China; 3 State Key Laboratory of Transducer Technology, Shanghai Institute of Microsystem and Information Technology, Chinese Academy of Sciences, Shanghai 200050, Chinawangyang@mail.sim.ac.cn (Y.W.);; 4 University of Chinese Academy of Sciences, Beijing 100049, China

**Keywords:** MEMS mirror, water immersible, piezoelectric, AlScN, cavitation

## Abstract

This paper presents a two-axis AlScN-based water-immersible MEMS mirror fabricated in an 8-inch MEMS process. Compared with other studies, this device has a larger optical aperture 10 mm in diameter. The resonant frequencies of the device are 1011 Hz in air and 342 Hz in water. The scanning angle reaches ±5° and ±2° at resonant frequencies in air and water, respectively. The cavitation phenomenon is observed when the device is operating in water, which leads the device to electrical failure. To address this issue, a device with reduced resonant frequencies—246 Hz and 152 Hz in air and water—is characterized, through which the bubbles can be effectively prohibited. This MEMS mirror could potentially be used in ultrasound and photoacoustic microscopy applications.

## 1. Introduction

MEMS mirrors have the advantages of small size, low power consumption, high resolution, fast response, and low cost [1,2,3,4]. They are widely used in the fields of laser projection [5], LIDAR [6], optical communication [7], and optical switching [8], where MEMS mirrors are mostly driven in air or a vacuum. Liquid-immersible MEMS mirrors have been developed for many applications, such as photoacoustic imaging [9], fluidic packaging [10], ultrasound imaging [11], etc. In photoacoustic imaging, MEMS mirrors are used to co-scan the ultrasound and optical beams to obtain the image. Due to the low propagation efficiency of ultrasound in air, MEMS mirrors are required to operate in water or other liquids. A conventional stepping-motor stage-based scanning system is often heavy and bulky, which makes it difficult to integrate into portable devices. To address this issue, MEMS mirrors are required to have a high scanning frequency to provide high pixel density for image reconstruction and a large optical aperture to obtain high-quality light and ultrasound beams.

The common driving modes of MEMS mirrors are electrostatic, electromagnetic, thermoelectric, and piezoelectric driving. Table 1 summarizes the cases of MEMS mirrors working in liquids with different driving methods in recent years. Kin et al. prepared an electromagnetically driven two-axis waterproof MEMS scanner using PDMS (polydimethylsiloxane) with a mirror size of 12 × 4 mm^2^ [12]. Due to greater damping, the resonant frequency decreased from 60 Hz in air to 45 Hz in water, while the scanning angle decreased from 8° to 4°. An in vivo photoacoustic image of a mouse ear was obtained by this MEMS scanner. Veljko et al. tested the working performance of MEMS mirrors driven by electrostatic combs in different liquids [10]. The experiment results showed that the scanning angle was directly proportional to the dielectric constant of the working medium. At the same voltage, the scanning angle in liquid with a dielectric constant of 2.73 was four times larger than that in air. Lei et al. fabricated an electrothermal mirror with a mirror size of 1 × 1 mm^2^. The optical angle was ±31° at a frequency of 100 Hz in air. The blood vessels of a human hand were reconstructed by the MEMS-based photoacoustic imaging system [13]. Duan et al. prepared a waterproof MEMS mirror using BoPET (biaxially oriented polyethylene terephthalate) and EN (elastomer nanocomposite) with a mirror size of 5 × 3 mm^2^ [11]. The mirror was driven by four permanent magnets. Its underwater operation required twice the driving voltage in air to achieve the same scanning angle. The resonant frequency was reduced from 295 Hz to 226 Hz. The MEMS mirror was used to achieve ultrasonic images of tungsten wires. Evidenced by the above driving methods, the electromagnetic mirrors are required to be equipped with bulky inductor coils and electromagnets to drive the mirror, making further integration and miniaturization difficult [14,15,16]. The electrostatic mirrors suffer from nonlinear electrostatic force, causing a complexity control system [17]. Due to the response time, the working frequency of electrothermal mirrors is usually limited [18]. Piezoelectric driving has the characteristics of high linearity, high scanning rate, high integration, and compatibility with semiconductor processes, making it a potential candidate to be widely used within all types of MEMS mirrors [19,20,21,22,23].

In this paper, we report on a two-axis water-immersible MEMS mirror based on AlScN piezoelectric thin film. The mirror consists of four identical cantilever beams and a large mirror plate 10 mm in diameter. Two-dimensional Lissajous scanning can be realized when four cantilever beams are driven simultaneously at resonant frequency. The performance of the mirror working in air and water is characterized. In addition, the cavitation phenomenon is observed when the mirror is working in water. A thicker mirror plate was proposed to reduce the resonant frequency and decrease the cavitation damage. Section 2 illustrates the driving principle, structure, simulation results, and fabrication process of the micromirror. Section 3 illustrates the characterization setup and working performance of mirror operation in air and water, respectively. Section 4 illustrates the cavitation damage caused by high-frequency vibration and the optimization of the device for this phenomenon.

## 2. Design and Fabrication

### 2.1. Mirror Design

**Figure 1:**
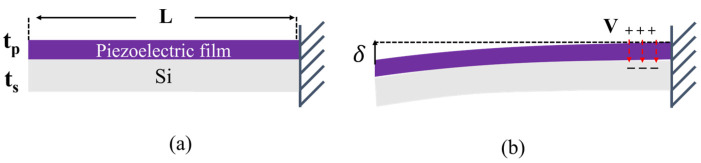
(**a**) Schematic diagram of piezoelectric cantilever beam; (**b**) cantilever beam bending caused by voltage driven.

The working principle of the piezoelectric micromirror is based on the inverse piezoelectric effect. As shown in Figure 1a, one silicon cantilever beam is covered with a piezoelectric layer. The beam length is *L*, while the thicknesses of the cantilever and the piezoelectric film are $t_{s}$ and $t_{p}$. When a voltage *V* is applied to the piezoelectric film, an electric field will be generated and thus induce a strain on the piezoelectric film. After that, the cantilever will bend and generate a displacement δ as shown in Figure 1b, which can be written as

(1) $$\delta =\frac{3s_{s}s_{p}t_{s}\left(t_{s}+t_{p}\right)L^{2}}{s_{s}^{2}t_{p}^{4}+4s_{s}s_{p}t_{s}t_{p}^{3}+6s_{s}s_{p}t_{s}^{2}t_{p}^{2}+4s_{s}s_{p}t_{s}^{3}t_{p}+s_{p}t_{s}^{4}}\cdot {V\cdot d}_{31}$$

where $d_{31}$ is the transverse piezoelectric coefficient of piezoelectric film, and $s_{s}$ and $s_{p}$ are the mechanical compliances of the cantilever and the piezoelectric film. According to Equation (1), when the substrate material and cantilever structure are given, the end displacement of the cantilever is proportional to the driving voltage and transverse piezoelectric coefficient. Hence, a piezoelectric material with high piezoelectric coefficient and high tolerable voltage is required to generate high displacement.

Piezoelectric materials used for driving actuators include PZT, AlN, and AlScN [24,25,26,27]. Due to the doping of the Sc element, AlScN has a higher piezoelectric coefficient than AlN, allowing for greater driving force and deflection. Compared with PZT, it has the benefits of higher linearity and higher tolerable operating voltage. Hence, AlScN is considered a more suitable material for driving micromirrors among the three piezoelectric materials. The AlScN thin film we used in the simulation model contains a Sc content of about 20%. The transverse piezoelectric coefficient is *d*_31,*AlScN*_ = −4.0 pm/V, and the longitudinal effective piezoelectric coefficient is *d*_33,*AlScN*_ = 9.9 pm/V. The results were obtained by characterizing Process Control Monitor (PCM) structures. Double-beam laser interferometry (DBLI) and Laser Doppler Velocimeter measurements were applied and combined with finite element method (FEM) simulations to extract the piezoelectric coefficient of the AlScN thin film [26].

**Figure 2:**
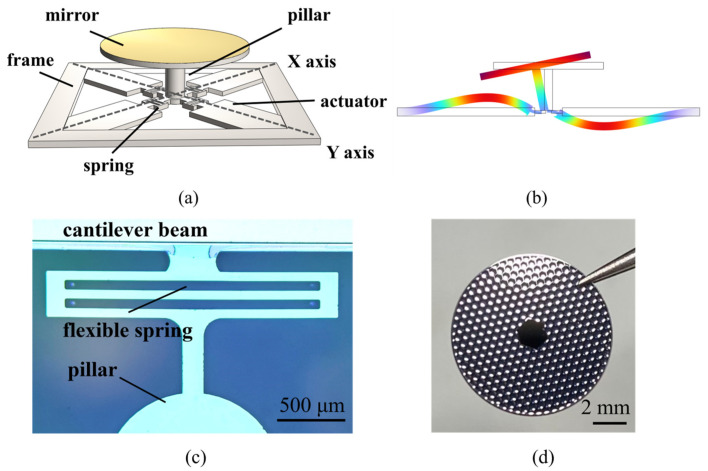
(**a**) Structure of MEMS mirror; (**b**) scheme of torsional motions along one axis; (**c**) photograph of the flexible spring; (**d**) photograph of the reinforced structure.

The structure of the MEMS mirror is shown in Figure 2a. The piezoelectric MEMS mirrors include two main kinds of structures: planar and stacked. The mirror plate of the planar structure is made of the top silicon of the actuator wafer, the thickness of which is usually limited. This device, with a large and thin mirror plate, is easily affected by water impact and results in mirror damage due to the limitation of the mirror plate [16]. Compared with the planar structure, the mirror plate of the stacked structure is made of another wafer besides the actuator wafer. The thickness and size of the mirror plate can be defined arbitrarily, which can effectively increase the reliability of the mirror plate. The micromirror consists of a gold-plated mirror, a supported frame, a pillar hidden in the mirror, and four cantilever beams. The mirror and actuators are prepared separately and finally bonded by flip-chip welding, which can control the alignment accuracy between the mirror plate and the actuator by 50 μm. A wafer-level bonding method is under development and will be applied to the device, which will control the alignment accuracy more precisely. The 10 mm optical aperture leads to an increase in mirror quality and a decrease in resonant frequency. To achieve a higher resonant frequency, a honeycomb-shaped reinforced rib structure was designed, intended to be etched into the back of the mirror plate to reduce the quality of the mirror plate and strengthen the resistance to water impact as shown in Figure 2d. A 1 μm AlScN thin film is deposited at the bottom of the cantilever beams to drive the mirror. The end of the cantilever beam is connected to the pillar by a flexible spring structure. Flexible spring structures can reduce the geometric nonlinearity of mechanical structures and enhance the flexibility of the beams. The photograph of the flexible spring structure is shown in Figure 2c, where one end of the flexible spring is connected to the piezoelectric cantilever beam and the other end is connected to the pillar. Compared with traditional MEMS mirrors, the mirror plate is designed to be 10 mm to provide a larger optical aperture for high-quality light and ultrasound beam steering. The main device parameters are listed in Table 2. The cross-section view of the MEMS mirror is shown in Figure 2b. As the opposite voltage is applied to a pair of AlScN films, a displacement in opposite directions is generated in the cantilever beams. The pillar bends and induces a tilting angle in the mirror. When four cantilever beams are driven simultaneously, 2D scanning can be realized.

**Figure 3:**
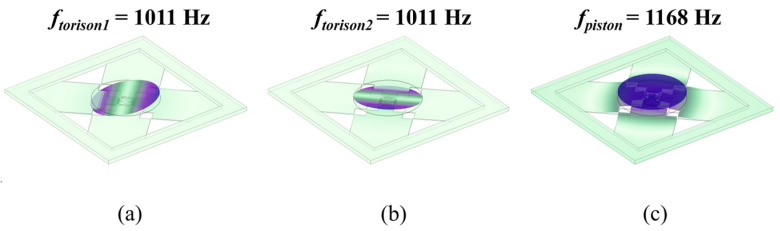
Resonant modes of MEMS mirror in air: (**a**) tip mode resonates at 1011 Hz; (**b**) tilt mode resonates at 1011 Hz; (**c**) piston mode resonates at 1168 Hz.

To seek an optimization design, parametric scanning was conducted to determine the main parameter values of the structure using COMSOL. The main scanning performance was simulated, as listed in Table 3. The resonant frequency of the structure was determined by modal analysis. The first three resonant modes and resonant frequencies of the device are shown in Figure 3. Due to having the same design parameters, the tip and tilt resonant modes of the device were the same at 1011 Hz, rotating around the X and Y axes, respectively. The third resonant mode of the device was piston mode, moving around the Z axis at 1168 Hz. Due to the great viscosity of water, the tip and tilt resonant frequencies in water were reduced to 297 Hz, and the piston resonant frequency was reduced to 354 Hz. The first two resonant modes will be used for laser and ultrasound scanning. The tilting resonant mode at 297 Hz meets the working requirement of ultrasound and photoacoustic imaging at 100–1000 Hz. The deflection angle of the device was calculated by the coupling of solid mechanics and electrostatic modules. The maximum tilting angle reached ±0.18° when applying ±90 V_DC_ in the actuators. The tilting angle of the mirror with AC driving was simulated; when the actuators are driven at resonant frequency, it reaches a ±5.5° optical angle at 90 V_AC_.

### 2.2. Fabrication and Assembly

**Figure 4:**
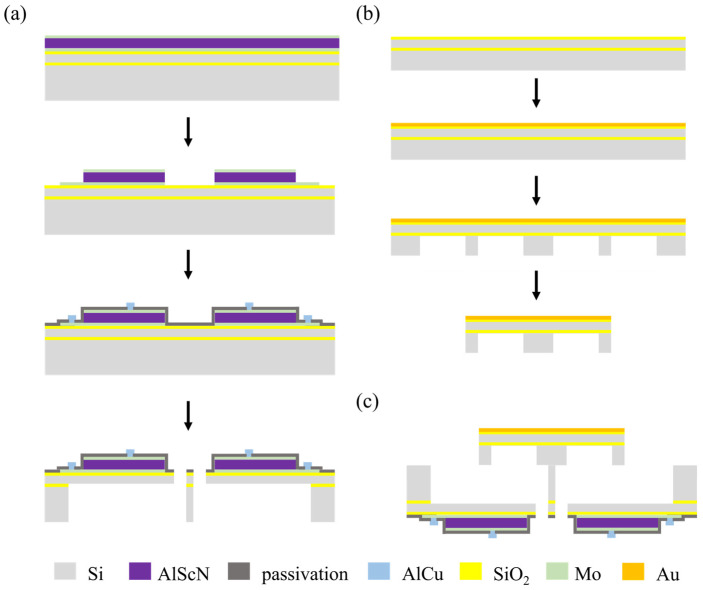
Fabrication process flow of MEMS mirror: (**a**) process flow for actuator wafer; (**b**) process flow for mirror wafer; (**c**) assembly of actuator and mirror.

**Figure 5:**
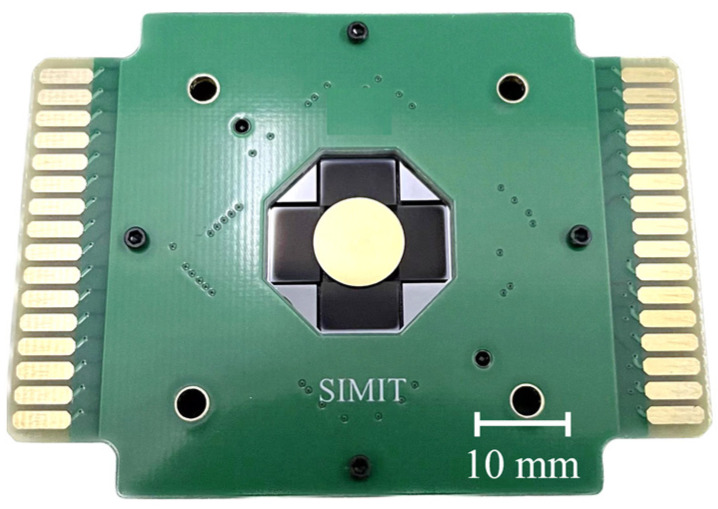
Photograph of assembled mirror fixed on PCB.

The device consists of four actuators and a gold-plated mirror plate. The actuator was fabricated as per the steps in Figure 4a: An SOI wafer with a 500 μm handle layer, a 1 μm buried oxide layer, and a 100 μm device layer were utilized as the starting material. Functional layers with the top electrode Mo (200 nm), the piezoelectric layer AlScN (1 μm), and the bottom layer Mo (200 nm) were deposited on the thermal oxide layer by magnetron sputtering. The functional layers were patterned and etched to form the top and bottom electrodes. The top and bottom Mo was etched with an ion beam, and the AlScN was wet-etched using 25% THMA (Tetramethylammonium hydroxide) at room temperature. On the top Mo layer, 200 nm silicon oxide was deposited by PECVD (Plasma-Enhanced Chemical Vapor Deposition) as the passivation layer and patterned to form the vias to the electrodes; a 1 μm AlCu layer was deposited by magnetron sputtering and patterned to form the electrical electrodes. The top silicon was patterned and etched to form the cantilever beams and springs through DRIE (Deep Reactive Ion Etching). Finally, the bottom silicon was etched through DRIE to release the cantilever beams and springs. Both of the DRIE steps stopped at the buried oxide layer to prevent over-etching. The mirror was fabricated as per the steps in Figure 4b: An SOI wafer with a 200 nm thermal oxide layer was prepared as the starting material. As the reflective layer, 30 nm Cr and 200 nm Au were sputtered on the wafer. A honeycomb-shaped reinforcement structure was patterned and etched into the bottom silicon to reduce the quality and strengthen the stiffness of the mirror plate. A laser cutting method was applied to release the mirrors from the wafer. Finally, the mirror and actuator were assembled by flip-chip bonding using epoxy, as shown in Figure 4c. The photograph of an assembled device is shown in Figure 5.

## 3. Characterization

**Figure 6:**
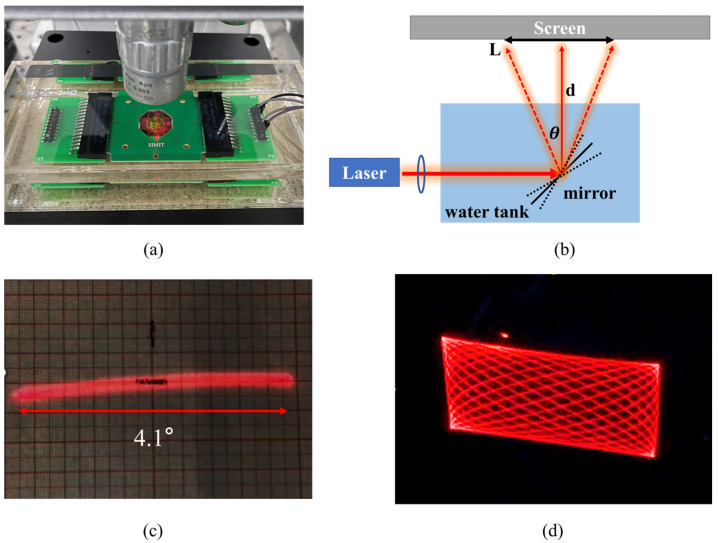
(**a**) Frequency spectrum test setup in water; (**b**) schematic diagram of the laser tracing method; (**c**) single-axis scanning result in air; (**d**) Lissajous scanning result in air.

The frequency spectrums of the proposed mirror in air and water were characterized using a Laser Doppler Velocimeter (Polytec MSA 500, Germany). The mirror was placed in pure water with electrical resistivity at 18.4 Mohm·cm to avoid electrical shorting; the characterization setup is shown in Figure 6a. The linearity of the MEMS mirror was characterized by an autocollimator. The amplitude of the DC driving voltage was limited from −90 V to 90 V to prevent the breakdown of the upper and lower electrodes. The scanning angle θ was measured at the resonant frequency using the laser tracing method in air and water, respectively. The characterization setup is shown in Figure 6b. The mirror was mounted in a water tank, the laser was projected onto the mirror through an adjustable diaphragm with an angle of 45°, and a screen was placed 70 cm in front of the mirror to receive the reflected laser. The scanning angle θ can be calculated with the following equation: θ=2×arctan (L/2d), where L represents the length of the reflected laser beam and d represents the distance between the mirror and the screen. The scanning result of single-axis driving at resonant frequency with 70 V is shown in Figure 6c.

**Figure 7:**
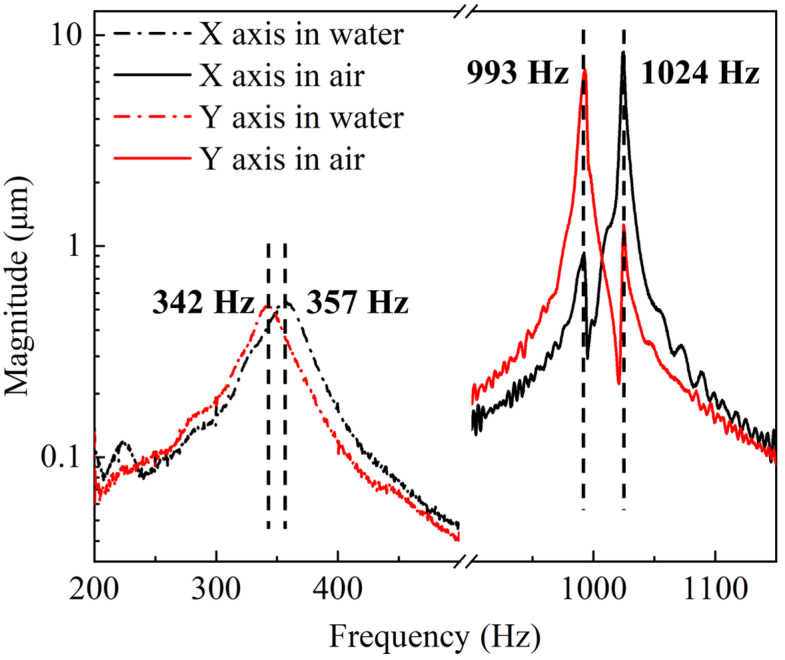
Resonance spectrums in air and water.

As shown in Figure 7, the resonant frequencies of the X and Y axes were determined to be 1024 Hz and 993 Hz in air. The difference between the two axes may be the nonuniformity of the DRIE of the four cantilever beams. The resonant frequencies of the X and Y axes were similar; when driving one of them in resonant frequency, another axis will be stimulated and coupled, causing a spurious peak appearing near the resonant frequency as shown in Figure 7. The measured resonant frequencies match well with the simulation result in air. When immersed in water, the resonant frequencies reduced to 342 Hz and 357 Hz due to a greater damping coefficient for water than air. Compared with the simulated results of 297 Hz, the characterized resonant frequencies were higher, probably due to the fact that the damping coefficient was not set accurately enough for water in the simulation model. The full width at half maximum (FWHM) and quality factor are important indicators for evaluating the performance of resonant mirrors. The FWHM is defined as the frequency bandwidth when the amplitude drops to 0.707 times the amplitude at the resonant frequency, which is commonly used to calculate the *Q* factor of the resonant devices [28]. The *Q* factor is used to describe the energy loss in a resonant device, which can be expressed as *Q =* $f_{0}/\Delta f$, where $f_{0}$ is the resonant frequency and Δf is the FWHM. A higher Q factor will lead to sharper frequency response, and the energy will be limited to the resonant frequency. The FWHMs of the mirror were determined to be 7 Hz and 34 Hz in air and water, respectively. The reduction in the FWHM and resonant frequency results in a much lower *Q* factor for the device in water than in air: from 146 to 11. The decrease in *Q* factor of the device working in water means that more energy was consumed by the device to overcome the resistance in water than in air. The Lissajous scanning pattern could be realized when simultaneously driving the X axis and Y axis at resonant frequency in air, as shown in Figure 6d.

**Figure 8:**
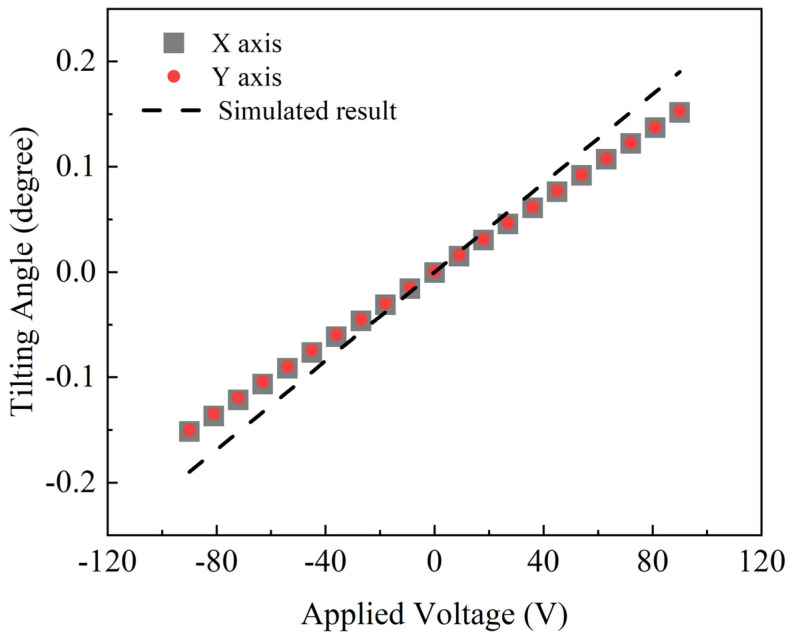
DC driving responses of two axes; simulated result represented by dashed line.

**Figure 9:**
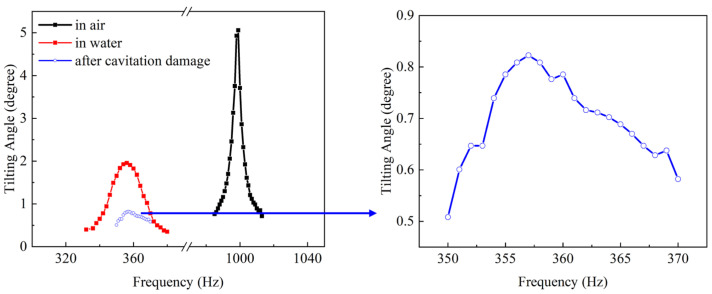
Frequency response characterization in air and water.

The relationship between the tilting angle and applied voltage under DC driving in the air is shown in Figure 8: the tilting angle of both the X and Y axes increased linearly with the driving voltage. Due to having the same design parameters, the curves of the two axes are almost identical, with great linearity to 99.3%. The tilting angle reached a maximum of ±0.15° at ±90 V_DC_. The measured angle was slightly smaller than the simulated angle in the dashed line, probably because of a deviation in the thickness and piezoelectric coefficient of the deposited piezoelectric film with the simulated model. The frequency responses of the device in air and water were characterized. Figure 9 shows the tilting angle of the X axis with an AC driving voltage of 90 V. When driven in resonant frequency, the scanning angle increased to 5° and 2° in air and water, respectively.

## 4. Cavitation Damage

**Figure 10:**
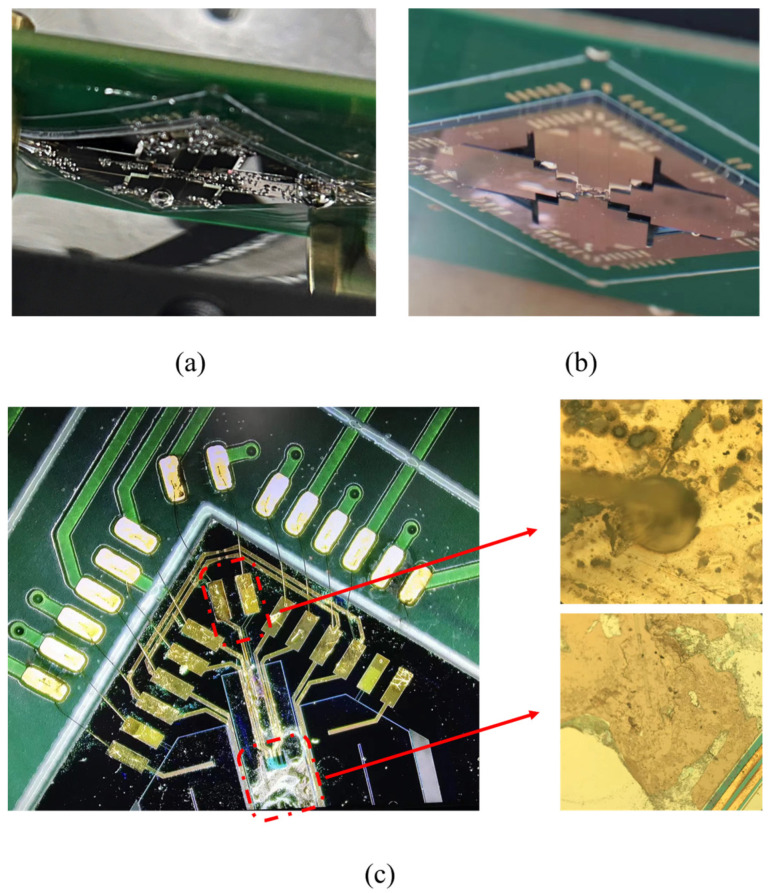
(**a**) Bubbles generated by normal device; (**b**) rare bubbles generated by thicker mirror device; (**c**) damage caused by cavitation in electrodes and cantilever beams.

Cavitation refers to the phenomenon of bubbles forming and bursting in liquid due to ultrasound or high-speed vibration, resulting in localized high temperature and pressure. This often occurs during the operation of centrifugal pumps and propellers, causing damage to the equipment [29,30,31]. When the device was driven at resonant frequency in water, it could be observed that fine bubbles were generated around the cantilever beams, as shown in Figure 10a. The bubbles generated by high-frequency vibration caused additional resistance to the motion of the cantilever, which may give another explanation for the lower tilting angle in water compared with the performance in air. After five minutes, the tilting angle at the resonant frequency under the same voltage was greatly reduced from the original 2° to 0.8°, as shown in Figure 9. This is because the localized high temperature and pressure generated by the bubbles caused irreversible damage to the cantilever beams. Figure 10c shows the cantilever beams damaged by bubbles after the device was operated in water. After ten minutes of operating in the water, the device stopped scanning. Cavitation etched holes at the electrode, causing conduction between the top and bottom electrode, and finally leading to the electric failure of the device. It can be seen that cavitation has an important effect on the reliability of the water-immersed MEMS mirror.

**Figure 11:**
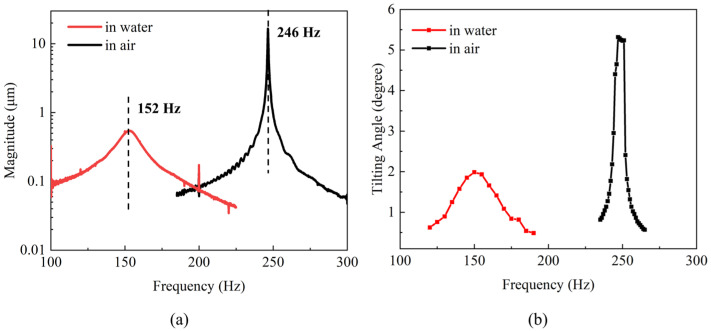
(**a**) Resonance spectrums of thicker mirror in air and water; (**b**) frequency responses of thick mirror device in air and water.

To avoid the cavitation phenomenon and its damage to devices, a thicker mirror wafer was prepared and affixed to the actuator to reduce the resonant frequency of the device. As shown in Figure 11a, the resonant frequency of the device with a thicker mirror was reduced from 1011 Hz to 246 Hz in air and 340 Hz to 152 Hz in water, respectively. To determine the scanning angle of the thicker mirror device at resonant frequency in air and water, the X axis was actuated at 90 V at the resonant frequency. As shown in Figure 11b, the device reached a 5.2° mechanical angle in air and reduced to 1.9° in water. As shown in Figure 10b, there were few bubbles generated and surrounding the actuators. This phenomenon verified the viewpoint that reducing operating frequency can effectively reduce air bubbles and the damage to the device. However, higher mirror quality resulted in higher mechanical loss, with the Q factor reduced to 33 and 5 in air and water compared with the results in Section 3. For a MEMS mirror working at resonant frequency, we want the *Q* factor of the device to be as high as possible. A low *Q* factor leads to more energy loss through various damping effects such as environment damping and mechanical loss. In addition, the lower *Q* factor leads the device to be more susceptible to external environmental interference. Minor external disturbances may cause the device to fail to stabilize in a resonant state. In addition to assembling a thicker mirror plate, a device with a larger mirror plate or cantilever beams with lower stiffness could be fabricated to reduce the resonant frequency and increase the scanning angle.

## 5. Conclusions

In conclusion, an AlScN-based water-immersible MEMS mirror with a large optical aperture of 10 mm has been developed and characterized. The mirror consists of four actuators and a mirror plate, both of which are fabricated with the standard MEMS process. The tilting angle of the mirror reaches ±0.15° at ±90 V at DC driving in air with a good linearity of 99.3%. The scanning angle at resonant frequency reaches ±5° and ±2° at ±90 V at AC driving in air and water, respectively. The cavitation phenomenon is found when the mirror is working in water at the resonant frequency, and the damage to the actuator could lead to mirror failure. A device with a thicker mirror plate was developed and characterized. Compared with ordinary devices, its resonant frequency was reduced from 1011 Hz to 246 Hz in air and 340 Hz to 152 Hz in water, respectively. Due to its lower resonant frequency, the bubbles could be effectively prohibited. The scanning angle of the thicker mirror device was ±5.2° and ±1.9° in air and water. In order to obtain better performance in water, a device with four lower-structure stiffness cantilever beams and a larger mirror plate will be developed to increase the scanning angle and decrease the resonant frequency in the future.

## Figures and Tables

**Table 1:** Summary of research into liquid-immersible MEMS mirrors.

Ref.	Mirror Size	Actuating Method	Chip size
[10]	D = 2 mm	electrostatic	5.2 × 5.2 × 0.6 mm^3^
[11]	5 × 3 mm^2^	electromagnetic	15 × 13 × 12 mm^3^ *
[12]	12 × 4 mm^2^ *	electromagnetic	15 × 15 × 15 mm^3^
[13]	1 × 1 mm^2^	electrothermal	2 × 2 × 3 mm^3^
[15]	6 × 4 mm^2^	electromagnetic	16 × 16 × 13 mm^3^
this work	D = 10 mm	piezoelectric	25 × 25 × 1 mm^3^

* Inferred from the figures in the paper.

**Table 2:** Main parameter values of the device.

Parameter	Value
mirror diameter	10 mm
actuator thickness	100 μm
mirror thickness	300 μm
frame width	2.5 mm
chip size	25 mm × 25 mm × 1 mm
chip mass	0.5 g

**Table 3:** Simulation results of the mirror.

Parameter	Value
resonant frequency in air	1011 Hz
resonant frequency in water	297 Hz
tilting angle under DC driving	±0.18°
tilting angle under AC driving at resonant frequency	±5.5°

## Data Availability

Data are available from the authors on request.
